# Regulation of apoptosis is impaired in atrophic gastritis associated with gastric cancer

**DOI:** 10.1186/s12876-017-0640-7

**Published:** 2017-06-29

**Authors:** R. Rosania, M. Varbanova, T. Wex, C. Langner, J. Bornschein, F. Giorgio, E. Ierardi, P. Malfertheiner

**Affiliations:** 1 Section of Gastroenterology, Department of Medical Sciences, University of Foggia, Foggia, Italy; 2 Department of Gastroenterology, Hepatology and Infectious Diseases, Otto-von-Guericke University, Magdeburg, Germany

**Keywords:** Atrophic gastritis (AG), Intestinal metaplasia (IM), Gastric cancer (GC), Helicobacter pylori

## Abstract

**Background:**

Gastric premalignant conditions, atrophic gastritis (AG) and intestinal metaplasia (IM) are characterized by an increase of proliferation and a reduction of apoptosis in epithelial cells. The epithelial cell kinetics in AG and IM in gastric mucosa adjacent to gastric cancer is still unclear. The aim of this study was to evaluate the epithelial cell turnover and expression of proliferation and apoptosis-related genes in gastric cancer (GC) and adjacent mucosa with atrophic gastritis or intestinal metaplasia (AG/IM GC+), as well as in atrophic gastritis or intestinal metaplasia mucosa of patients without GC (AG/IM GC-) and in control biopsy samples of non-transformed gastric mucosa (Control).

**Methods:**

We selected 58 patients (M: F = 34:24; age range 20-84 years, median 61.06 years) with 4 well defined histological conditions: 20 controls with histological finding of non-transformed gastric mucosa, 20 patients with AG or IM (AG/IM GC-), and 18 patients with intestinal type gastric adenocarcinoma (GC) and AG or IM in the adjacent mucosa (3 cm from the macroscopic tumour margin, AG/IM GC+). We performed an immunohistochemical staining of Ki67 and TUNEL and quantitative RT-PCR to determine the expression of PCNA and Bax/Bcl-2.

**Results:**

The immunohistochemical expression of Ki67 and TUNEL in AG/IM GC- was significantly increased compared to not transformed gastric mucosa (*p* < 0.0001) but not compared to AG/IM in gastric mucosa adjacent to GC. Levels of Bcl-2 were reduced in GC and AG/IM GC- compared to controls as well as in AG/IM GC- compared to AG/IM in mucosa adjacent to GC+ (*p* < 0.05). Proliferation and apoptosis markers did not correlate with *H.pylori* status in our study population.

**Conclusions:**

In AG/IM associated with GC, no significant changes in the epithelial cell turnover were detected. Decreased Bcl-2 gene expression signified atrophic gastritis and IM in presence of cancer, as well as intestinal type gastric adenocarcinoma.

## Background

For intestinal type gastric cancer has been proposed a well-defined multistep model [1] that comprises gastric precancerous conditions, atrophic gastritis (AG) and intestinal metaplasia (IM). A common feature of malignancies is an alteration of cell turnover leading to cell hyperproliferation and to deregulation of apoptosis [2, 3]. During gastric carcinogenesis, epithelial cell proliferation shows an increasing trend from chronic gastritis to gastric cancer linked to *Helicobacter pylori* infection [4–6]. Apoptosis is increased in chronic gastritis but decreases when chronic gastritis progresses to IM or AG [7–9]. This discrepancy of proliferation and apoptosis is reversed after *H. pylori* eradication [10–12].

The balance of the epithelial cell between proliferation and apoptosis is a complex process controlled by a large number of genes, including the proliferating cell nuclear antigen (*PCNA*) and members of the Bcl-2 family such as *Bax* and *Bcl-2*. The *PCNA* gene plays a key role in DNA replication, DNA repair and cell cycle control [13]. The *Bax* gene is a tumour suppressor gene encoding the *Bax* protein that promotes apoptosis [14]. *Bcl-2* is an anti-apoptotic oncogene encoding a protein that antagonizes the function of *Bax* [15], and its overexpression during carcinogenesis leads to an accumulation of aberrant cells by preventing them from undergoing apoptosis. *Bcl-2* was initially recognized as an oncogene in follicular lymphoma [16]; however, some recent reports suggest contradictory effects of this protein in different cancer entities [17–19]. The proliferation of epithelial cells can be assessed by analysing Ki67, which is detected only during the growth and synthesis phase but not during the resting phase of cell cycle [20]. Similarly, terminal deoxynucleotidyl transferase dUTP nick end labeling (TUNEL) is a common method to reveal DNA fragmentation resulting from apoptotic signalling cascades [21]. The epithelial cell turnover and the expression of the regulatory cell cycle genes in atrophic gastritis or intestinal metaplasia in the mucosa adjacent to gastric cancer has not been investigated.

The aim of the present study was to evaluate the expression of genes involved in the control of proliferation and apoptosis, analysing the transcript level of *Bax*, *Bcl-2* and *PCNA* in gastric cancer (GC) and in atrophic gastritis and/or intestinal metaplasia in adjacent mucosa to gastric cancer (AG/IM GC+), as well as in atrophic gastritis and/or intestinal metaplasia in patients without gastric cancer (AG/IM GC-) and in control biopsy samples of non-transformed gastric mucosa from subjects with functional dyspepsia. We hypothesized an impaired control of epithelial cell kinetics in gastric cancer and adjacent mucosa with atrophic gastritis and/or intestinal metaplasia (AG/IM GC+). Secondarily we performed an immunohistochemical evaluation of Ki67 and TUNEL in all the 4 histological defined groups: GC, AG/IM GC+, AG/IM GC- and control to assess the epithelial cell active in the proliferative phase of the cell cycle and in the apoptosis.

## Methods

### Study design and patients

From a cohort of patients, previously enrolled, we selected subjects with 4 histological defined conditions. The control group comprised 20 patients (M 11: F 9, mean age 55.8 ± 15.3 years, range 22-75 years) who underwent upper gastrointestinal endoscopy for functional dyspepsia that did not show any histological sign of premalignant or malignant transformation. We also enrolled 20 patients (M 10: F 10, mean age 64.2 ± 14.6 years, range 25-81 years) with histological diagnosis of atrophic gastritis and/or intestinal metaplasia (AG/IM GC-). Inflammatory and preneoplastic changes of the gastric mucosa have been graded according to the updated Sydney system [22].

The study protocol was conducted according to the declaration of Helsinki and approved by the ethical board of the Otto-von-Guericke University. All subjects provided written informed consent before entering the study. The following exclusion criteria were applied: other types of cancer (except gastric cancer), operated stomach, severe renal or hepatic impairment, irradiation of the upper abdomen, immunosuppressive therapy, oral anticoagulation and antibiotic therapy in the last 2 weeks before entering the study. All individuals underwent upper gastrointestinal endoscopy at the Department of Gastroenterology, Hepatology and Infectious Diseases, Otto-von-Guericke University. In patients without gastric cancer, biopsies for molecular analysis were retrieved and immediately snap-frozen in liquid nitrogen and stored at −80 °C until use. 5 biopsies (2 antrum, 2 corpus and 1 incisura angularis) were taken for histological evaluation and immediately fixed in buffered formalin. Finally one biopsy was obtained from both antrum and corpus for rapid urease test (HUT®, Astra Zeneca, Wedel, Germany). *H. pylori* status was determined by rapid urease test, histology and microbiology. In patients with first diagnosis of gastric cancer, biopsies were taken from the tumour itself and from peritumoral mucosa (3 cm from the macroscopic tumour margin) and immediately snap-frozen in liquid nitrogen and stored at −80 °C. Then biopsies from the tumour, the peritumoral mucosa (3 cm from border of tumour) and the tumour free zone were retrieved for histological evaluation. We selected a group of 18 patients (M 13: F 7, mean age 54.7 ± 7.72 years, range 43-84 years, 15 patients (83%) *H. pylori* positive) with histological diagnosis of intestinal type gastric cancer and with atrophic gastritis and/or intestinal metaplasia in adjacent mucosa. Tumor adjacent samples exhibiting AG and IM (AG/IM GC+) and carcinoma samples (GC+) were analyzed as two separate groups (Table 1). None of the patients with gastric cancer had chemotherapy, radiotherapy or gastric surgery prior to endoscopy.

**Table 1:** Study population characteristics

	*Controls*	*AG/IM GC-*	AG/IM GC +*	*GC*	*P*
*N*	20	20	18	18	NS
*Age (years ± SD)*	55.8 ± 15.3	64.2 ± 14.6	54.7 ± 7.72	54.7 ± 7.72	<0.05
*Sex (M), (%)*	11 (55%)	10 (50%)	13 (72%)	13 (72%)	NS
*Hp Status, positive (%)*	0	12 (60%)	15 (83%)	15 (83%)	<0.05

We selected patients with 4 histological defined conditions: gastric cancer intestinal type (GC), atrophic gastritis with intestinal metaplasia in adjacent mucosa to gastric cancer (AG/IM GC+), atrophic gastritis with intestinal metaplasia in patients without gastric cancer (AG/IM GC-) and control biopsy samples of non-transformed gastric mucosa. Parameters were analyzed using ANOVA, t Student for unpaired data and Chi-square-test for categorical data, (significance *p* < 0.05, NS: not significant). *in patients with GC, we considered as separated group samples from tumor and from adjacent mucosa

### Extraction of total RNA, cDNA synthesis and quantitative RT-PCR

Due to different periods of enrollment, two different kits have been used for RNA extraction. The RNeasy plus Universal Mini Kit (Qiagen, Hilden, Germany) was used to extract RNA in controls and in patients with atrophic gastritis and intestinal metaplasia (AG/IM GC-). For gastric cancer patients was used the RNeasy Mini Kit (Qiagen, Hilden, Germany).

In both kits a single biopsy was homogenized in 900 μl QIAzol Lysis using tissue raptor (QIAGEN, Hilden, Germany). Afterwards, the RNA was purified using the RNeasy kit according to the manufacturer’s instruction. Finally, the RNA was eluted in 70 μl RNase-free water. 8 μl of each sample was used to check RNA quality by agarose gel electrophoresis and determination of RNA concentration via UV-spectroscopy. The cDNA synthesis was performed with 2000 ng for controls and AG/IM patients and with 500 ng of total RNA in the gastric cancer group within a 40 μl reaction volume by AMV reverse transcriptase (Promega, Mannheim, Germany) with random hexanucleotides (Boehringer, Mannheim, Germany) as described previously [23]. PCNA, Bax, Bcl-2 and β-actin transcript levels were determined by quantitative real-time RT-PCR using a CDX96-Cycler (BioRad, Munich, Germany). The 30 μl reaction mixture contained 13.4 μl RNase-free water, 15 μl HotStarTaq-Sybr. Green, 0.2 μl of both primers (50 μM) and 1.2 μl cDNA. Initial denaturation and activation of Taq-polymerase at 95 °C for 15 min was followed by 40 cycles as described in Table 2. Primers used and the size of expected PCR fragments are also listed in Table 2. Amplification of genomic cDNA was excluded due to usage of intron spanning regions for the primers. The fluorescence intensity of the double-strand specific SYBR-Green I, reflecting the amount of actually formed PCR-product, was read real-time at the end of each elongation step. Initial template mRNA amounts were calculated by the ΔCt-algorithm. Final results are expressed as arbitrary units (a.u.) and represent ratios between target gene (PCNA, Bax, Bcl-2) and β-actin transcript amounts.

**Table 2:** RT-PCR primers for each gene

*Gene*	*Primer forward*	*Primer reversed*	*Product Size*
*ß-actin*	5′-catgccatcctgcgtctgcacc-3′	5′-acatggtggtgccgccagagaca-3′	400 bp
*Bax*	5′-ccaaggtgccggaactgatc-3′	5′-aacacagtccaaggcagctgg-3′	212 bp + 780 bp
*Bcl-2*	5′-gaaccggcacctgcacacctg-3′	5′-aagctcccaccagggccaaac-3′	143 bp + 700 bp
*PCNA*	5′-tctgagggcttcgacacctacc-3′	5′-acgtgcaaattcaccagaaggc-3′	279 bp +8,5 kb

The standard condition for real time were as follow: 95 °C: 15 min (95 °C: 30s 60°C: 72 °C: 30s), 40 cycles; 72 °C: 5 min (annealing time varied between genes); size of the amplification product is given as base pairs (bp)

### Immunohistochemical staining of Ki67

Ki67 staining was realized using a monoclonal antibody (clone MIB-1, DAKO, Glostrup, Denmark). The sections were de-waxed in xylene and then rinsed in alcohol and graded alcohol/water mixtures. 3% hydrogen peroxide was then applied to block endogenous peroxidase activity. The sections were subsequently treated in a microwave oven twice for 5 min in citrate buffer (pH 6.0) at high power (750 W). After blocking with 10% goat serum, the primary antibody was applied and Ki67 rabbit polyclonal antibody diluted 1: 100 (DAKO, Carpinteria, CA) was used with incubation time for 1 h at room temperature. After rinsing, the secondary anti-rabbit peroxidase-conjugated antibody (EnVision,DAKOA/S, Glostrup, Denmark), was applied to the sections according to the manufacturer’s instructions (incubation for 40 min at room temperature). Peroxidase activity was detected by application of DAB (Vector Laboratories, Burlingame, CA, USA). The sections were then counterstained with hematoxylin, dehydrated, cleared and mounted.

### Immunohistochemical staining of TUNEL

Paraffin-embedded samples were analyzed for DNA fragmentation using a TUNEL assay with the In Situ Cell Death Detection Kit (Roche Molecular Biochemicals, Indianapolis, IN, USA). According to the manufacturer’s instruction, 3-μm-thick paraffin sections of the biopsies were deparaffinized in xylene and rehydrated in decreasing concentrations of ethanol. Sections were rinsed in distilled water and incubated in 3% hydrogen peroxide in methanol for 5 min to block endogenous peroxidase activity. Tissue sections were then incubated in 20 μg/ml proteinase K (DAKO Corporation, Carpinteria, CA, USA) for 15 min, washed with PBS, incubated in equilibration buffer and then in TdT enzyme solution in a humidified chamber at 37 °C for 60 min. The sections were subsequently rinsed in PBS, and then incubated with streptavidin-peroxidase (Vector Laboratories, Burlingame, CA, USA) conjugate for 30 min. Peroxidase activity was detected by application of DAB. Apoptotic activity was identified by a dark brown nuclear stain observed under a light microscope.

### Cell count and labeling index

Ki67 and TUNEL positive cells were counted and counted both in the whole foveolae and in the upper third of the foveolae. The count was performed only in well-oriented crypts of the upper half of the mucosal thickness using a television camera with a grid placed on the screen. The count was stopped when columnar cells disappeared and glandular cells with a bubbled cytoplasm were observed. The nuclei completely and uniformly stained red-brown were considered as positive. To evaluate the Ki67 or TUNEL labeling index (LI score) in areas of intestinal metaplasia according to Filipe [24]. 2–6 fields were chosen and well-oriented foveolae representing the lesion were selected. A mean of 3 crypts/field, depending on the extent of the lesion were counted.

### Statistical analysis

Data were entered into a database using the Microcal Origin™ 8.0 program package (Northampton, MA, USA). Statistical analysis of gene expression and immunohistochemical LI score between all the groups was performed with the Kruskal-Wallis test. In case of significant results, we performed post-hoc pair wise comparisons using the non-parametric Mann-Whitney U test with a Bonferroni correction to account for multiple comparisons. Correlations or associations between paired gene expression and LI score and *H. pylori* status or histological parameters were calculated by Spearman’s rank correlation test. All tests were applied two-sided with a significance level of *p* < 0.05.

## Results

### Comparison of demographic data

As shown in Table 1, patients with AG and IM in patients without gastric cancer were significantly older than the controls (Student’s t-test for unpaired data, *p* = 0.04) and the patients with intestinal type gastric cancer (Student’s t-test for unpaired data, *p* = 0.01). The prevalence of *H. pylori* infection was higher in intestinal type gastric cancer (GC, 83%) than in AG and IM in patients without cancer (60%, Chi square test, *p* < 0.05).

### Expression of apoptotic and proliferative related genes

**Fig. 1:**
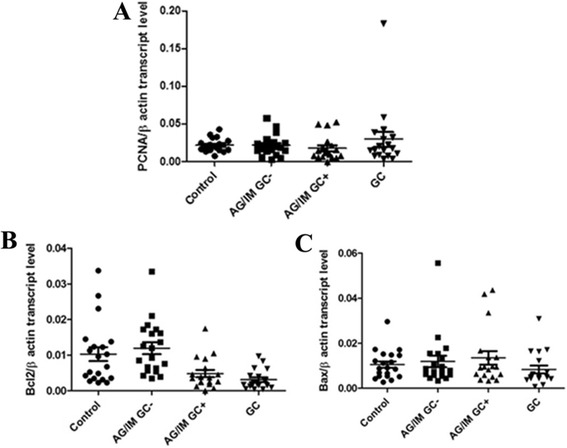
Gene expression of PCNA, Bcl-2 and Bax in the study groups. **a** PCNA transcript level was expressed in all the population but did not differ between the 4 histological defined groups (Kruskal Wallis H = 4.18, *p* > 0.05). **b** Bcl-2 showed a significant trend toward decreasing from control to gastric cancer (Kruskal-Wallis H = 27.16, *p* < 0.05) and demonstrated a significant reduction in AG/IM GC- compared to control (Mann-Whitney’s U test *p* < 0.05), in AG/IM GC- compared to AG/IM GC+ (Mann-Whitney’s U test *p* < 0.05) and in GC compared to AG/IM with and without cancer and in control ((Mann-Whitney’s U test *p* < 0.05). **c** Bax expression did not differ in the study population (Kruskal Wallis H = 4.07, *p* > 0.05)

Transcript levels of *PCNA* were detectable in all study samples (Fig. 1a), but did not differ between the groups (Kruskal-Wallis H = 4.18, *p* > 0.05). Considering the histological parameters of Sydney System score [22], PCNA transcript gene level showed a correlation only with the grade of inflammatory chronicity in AG/IM GC- (Spearman test *r* = 0.56, *p* < 0.01) but no significant correlations were found in the analysis with the other groups or with the grade of inflammatory activity (Spearman test *p* > 0.05). Moreover, there was no significant association between PCNA transcript levels and positivity for *H. pylori* infection (Spearman test *p* > 0.05). The expression of anti-apoptotic gene *Bcl-2* was analysed in all conditions and showed a significant decrease from controls to GC (Kruskal-Wallis H = 27.16, *p* < 0.05). *Bcl-2* gene expression in controls was significantly increased than in AG/IM GC+ and GC (Mann-Whitney’s U test *p* < 0.05; Fig. 1b). Moreover, *Bcl-2* expression in AG/IM GC+ was significantly decreased compared to AG/IM GC-. *Bcl-2* gene transcript level did not show any correlation with the grade of activity and chronicity of atrophic gastritis and intestinal metaplasia neither with the positivity for *H. pylori* infection (Mann U Whitney’s U test *p* > 0.05). The expression of *Bax*, a pro-apoptotic gene, was detected in all steps of carcinogenesis (Fig. 1c) but did not show any significant difference between the groups (Kruskal-Wallis H = 4.07, *p* > 0.05). Bax transcript level did not show any association neither with *H. pylori* status or other inflammatory parameters.

### Immunohistochemical assessment of apoptotic and proliferative marker

**Fig. 2:**
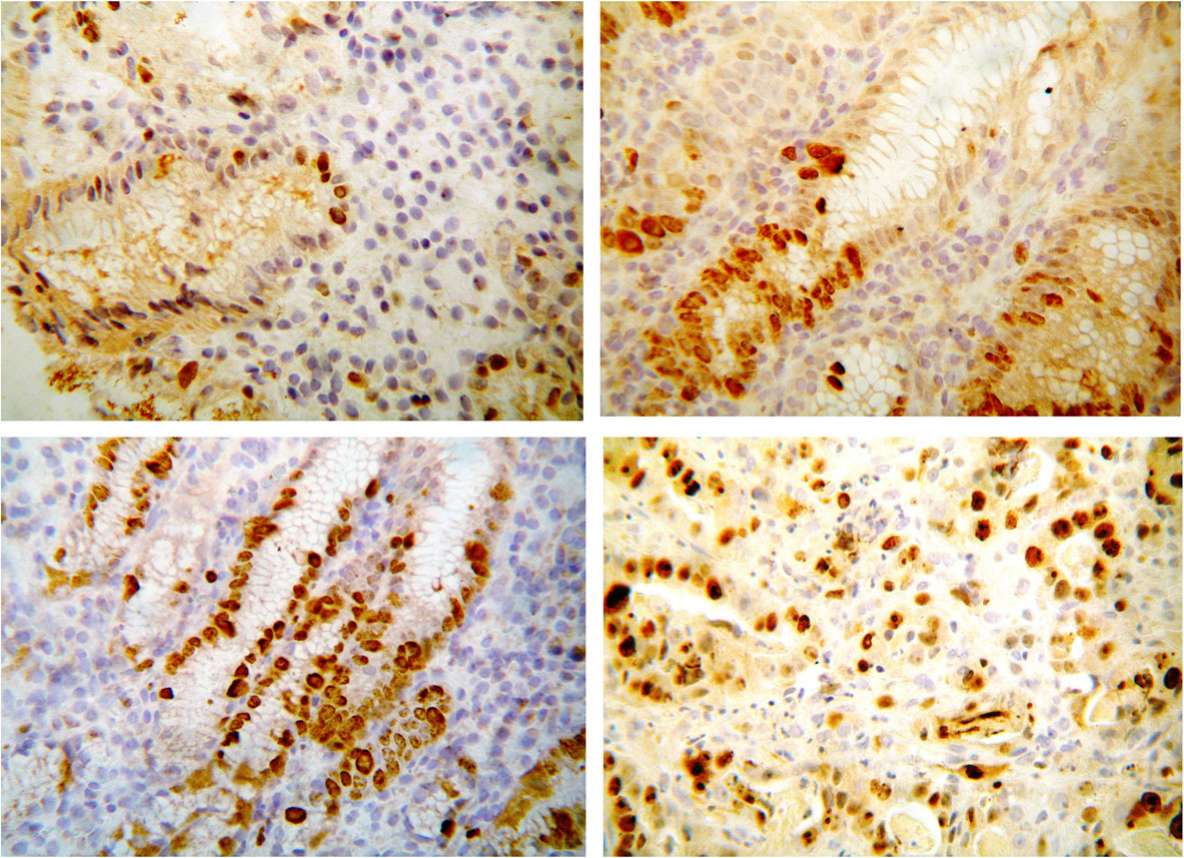
Immunohistochemical Ki67staining in AG with IM in presence of cancer. KI67 staining in normal gastric mucosa was limited to epithelial cell of glandular neck (*upper left*) but was increased in presence of inflammatory (*upper right*), metaplastic (*down left*) and neoplastic changes (*down right*). The epithelial staining of Ki67 was significantly different in all groups (Kruskal-Wallis H = 57.31 *p* = 0.0001) with a progressive increase from controls to AG and IM in patients without gastric cancer (Mann-Whitney’s U test *p* = 0.0001) and to intestinal type gastric cancer (Mann-Whitney’s U test *p* = 0.001)

The epithelial staining of Ki67 was significantly different in all groups (Kruskal-Wallis H = 57.31 *p* = 0.0001) with a progressive increase from controls to AG and IM in patients without gastric cancer (Mann-Whitney’s U test *p* = 0.0001) and to intestinal type gastric cancer (Mann-Whitney’s U test *p* = 0.001). We did not find any significant difference in the Ki67 staining between AG/IM GC- and atrophic gastritis with intestinal metaplasia in mucosa adjacent to intestinal type gastric cancer (Mann-Whitney’s U test *p* = 0.05). The Ki67 immunohistochemical staining was limited in the control group to epithelial cells of the “glandular neck” and showed an increased staining in presence of inflammatory, metaplastic and neoplastic changes (Fig. 2). On the other hand, any change of Ki67 LI was reported in the samples positive for Helicobacter pylori infection.

**Fig. 3:**
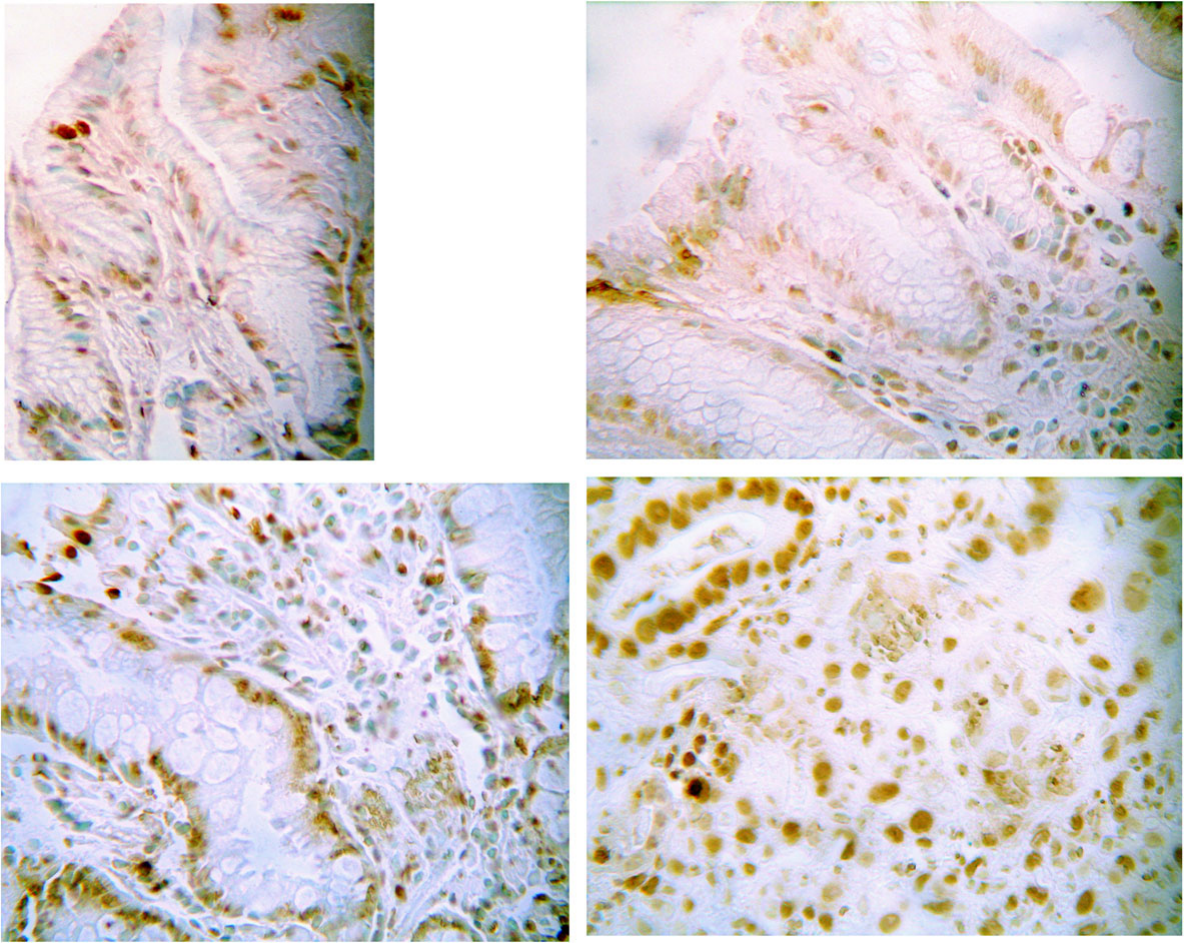
Immunohistochemical TUNEL staining in normal mucosa, AG/IM without cancer and GC. TUNEL was expressed in non-transformed gastric mucosa (*upper left*) but its staining was significantly increased in presence of atrophic (*upper right*) and metaplastic (*down left*) or neoplastic changes of the mucosa (*down right*). The TUNEL staining differed significantly between the analysed groups (Kruskal-Wallis H = 37.11, *p* = 0.0001) and increased progressively from controls to AG and IM in patients without gastric cancer (Mann-Whitney’s U test *p* = 0.001) and to intestinal type gastric cancer (Mann-Whitney’s U test *p* = 0.007)

The TUNEL assay differed significantly between the analyzed groups (Kruskal-Wallis H = 37.11, *p* = 0.0001) and increased progressively from controls to AG and IM in patients without gastric cancer (Mann-Whitney’s U test *p* = 0.001) and to intestinal type gastric cancer (Mann-Whitney’s U test *p* = 0.007). We did not find any significant difference in the epithelial TUNEL staining between AG/IM in patients without gastric cancer and atrophic gastritis with intestinal metaplasia in adjacent mucosa to GC (Mann-Whitney’s U test *p* = 0.32). TUNEL was expressed in non-transformed gastric mucosa but its staining was significantly increased in presence of atrophic and metaplastic or neoplastic changes of the mucosa (Fig. 3). The TUNEL LI showed an increasing staining on the samples positive for Helicobacter pylori infection.

## Discussion

An impaired balance between proliferation and apoptosis was detected in gastric cancer and in AG or IM in gastric mucosa adjacent to cancer. On a molecular level the *Bcl-2* gene expression was decreased in GC compared to AG/IM GC- and controls. Although it may appear paradoxical, this finding is supported by strong evidence that *Bcl-2* and *Bcl-2 L10* (*Bcl-2* family member) expression inhibits growth in solid tumors and gastric cancer in particular [25, 26]. *Bcl-2* is widely known to be an antiapoptotic proto-oncogene involved in carcinogenesis and is currently assessed for its use as a therapeutic target for B cell non-Hodgkin’s lymphoma. The gene product is physiologically found in bone marrow progenitor cells, intestinal and epidermal stem cells, while absent in differentiated tissue [27]. *Bcl-2* is detectable in chronic gastritis [28, 29] but under expressed in advanced and less differentiated GC [30–32]. We assume a distinct function of the *Bcl-2* protein in neoplastic gastric mucosa as discussed by Pietenpol et al. [25], who observed a profound growth suppression when *Bcl-2* was transfected in colorectal, lung adenoma and brain glioblastoma cell lines. In a study of 501 Chinese GC patients after D2 gastrectomy, the less common *Bcl-2* positive gastric cancers (22%) were significantly associated with better survival rates [32]. *Bcl-2* expression was identified as an independent prognostic factor among p53, *bax* and *c-myc* [33]. Combined staining of *Bcl-2* expression with Ki67 LI in GC clearly showed a negative correlation, indicating that Bcl-2 positive GC cells were in a non-proliferative state [34, 35]. Taken together, evidence suggests that *Bcl-2* probably acts as a tumor suppressor gene in gastric cancer.

Notably, we observed significantly decreased *Bcl-2* gene expression in AG and IM in gastric mucosa adjacent to GC compared to AG and IM in patients without GC. In tumor adjacent mucosa, *Bcl-2* was found in lower levels compared to GC [36] but to our knowledge no study has so far focused on *Bcl-2* in atrophic gastritis and intestinal metaplasia in tumor adjacent mucosa. Our data demonstrate that lack of *Bcl-2* expression, which is common in gastric cancer, is also frequently found in atrophic gastritis and intestinal metaplasia in mucosa adjacent to GC and may indicate a field of cancerisation, in which gastric cancer can arise. We confirmed *Bax* and *PCNA* gene expression in all groups [37, 38] but without any significant changes between the four histological defined conditions. *Bax* expression was analyzed in gastric adenocarcinoma and non-transformed peritumoral mucosa and no significant difference was found [36]. Similarly, in our study *Bax* expression did not vary in atrophic gastritis with intestinal metaplasia in adjacent mucosa and in GC. *PCNA* expression is generally used as a molecular proliferation marker, which was reported to increase during progression to gastric cancer [38, 39], showing a significant correlation with acute and chronic inflammation [9]. *PCNA* and *Bax*, although expressed at various steps of gastric carcinogenesis, do not show differential expression between different levels of proliferation in progression to gastric cancer.

As expected, the staining of Ki67 and TUNEL showed an increasing trend from controls to GC but did not change between AG and IM in patients without gastric cancer and in AG and IM in adjacent mucosa to GC. Also, Ki67 staining did not show correlation with *H. pylori* infection or any parameter of inflammation, in contrast to other reports [35, 36]. However, we were able to show increased apoptosis in gastric epithelial cells in H. pylori infection as reported repeatedly [9, 39]. The number of apoptotic epithelial cells is increased from 2% in normal mucosa to 8-17% in gastritis. This process is mediated by induction of CD 95 and TNF receptor by *H. pylori* itself or cytokines from chronic inflammation [8, 40]. Triantafyllou et al. investigated Ki67 and TUNEL as apoptosis and proliferation markers in tumor adjacent tissue to gastric cancers (intestinal type) and reported no significant difference between tumor and surrounding mucosa [34, 41, 35]. On the contrary, we found a pronounced increase in Ki67 LI and apoptosis levels in cancer compared to adjacent AG/IM. This might be due to the fact that all GC in our study were in advanced stages (data not shown), where significantly higher apoptosis levels were found.

## Conclusions

Despite the limitation by a relatively small number of patients, we identified a lack of Bcl-2 expression as a potential marker of malignant transformation in gastric mucosa. Our data provide an insight into the peritumoral atrophic gastritis as a field of cancerisation. This was the first study to our knowledge focusing on epithelial cell kinetics in atrophic gastritis with intestinal metaplasia in adjacent mucosa to GC. Further investigation is needed to better explain the delicate interplay of apoptosis and proliferation regulating factors and their distinct function in gastric carcinogenetic cascade.

## Abbreviations

AG: Atrophic gastritis
AG/IM GC-: Atrophic gastritis or intestinal metaplasia mucosa of patients without GC
AG/IM GC +: Adjacent mucosa with atrophic gastritis or intestinal metaplasia
GC: Gastric cancer
IM: Intestinal metaplasia
